# Determinants of sexually transmitted infections among female sex workers in Ethiopia: a count regression model approach

**DOI:** 10.3389/fpubh.2023.1190085

**Published:** 2023-08-04

**Authors:** Feyiso Bati Wariso, Jemal Ayalew, Ammar Barba, Birra Bejiga Bedassa, Gemechu Gudeta Ebo, Jaleta Bulti Tura, Mohammed Rameto, Wudinesh Belete Belihu, Derbachew Asfaw, Minilik Demissie Amogne, Lemessa Negeri, Sileshi Lulseged, Saro Abdella Abrahim

**Affiliations:** ^1^Ethiopian Public Health Institute, Addis Ababa, Ethiopia; ^2^College of Natural Sciences, Wollo University, Dessie, Ethiopia; ^3^College of Health Sciences, Addis Ababa University, Addis Ababa, Ethiopia

**Keywords:** serology, epidemiology, sexually transmitted infections, female sex workers, hurdle poison regression model

## Abstract

**Background:**

Sexually transmitted infections (STIs) remain a major public health problem worldwide, with the burden of these infections being high among female sex workers (FSWs), who are often not aware of their infection status. This study aimed to determine the factors that are associated with the number of STIs among FSWs in Ethiopia.

**Methods:**

A cross-sectional bio-behavioral study involving respondent-driven sampling (RDS) was conducted among 6,085 FSWs in 16 towns in Ethiopia. The hurdle Poisson regression model was fitted using STATA Version 16.2. The incident rate ratio and adjusted odds ratio with a 95% confidence interval were employed to show the strength and direction of the association. A *p*-value of ≤0.05 was used as a threshold for statistical significance.

**Results:**

At least one STI was identified in 1,444 (23.64%) of the FSWs. Age group 35–49 years [IRR = 2.32; 95% CI (1.43, 3.74)], forced first sex [IRR = 1.32; 95% CI (1.01, 1.74)], condom breakage [IRR = 1.32; 95% CI (1.01, 1.74)], and a history of depression [IRR = 1.55; 95% CI (1.12, 2.18)] increase the number of STIs. FSWs aged 25–34 years [AOR = 2.99; % CI (2.54, 3.52)] and 35 = 59 years [AOR = 8.05; % CI (6.54, 9.91)], who were selling sex for 5–10 years [AOR = 1.30; 95% CI (1.1, 1.55)], and above 11 years [AOR = 1.21; 95% CI (1.03, 1.43)] were more likely to get STIs.

**Conclusion:**

STIs are common in Ethiopia. The covariates age, educational status, monthly income, condom failure, age at the first sexual encounter, and long duration of sexual practice are significant predictors of STIs. Health interventions among FSWs need to include awareness generation about the prevention and control of STIs and address the determinants identified in this analysis.

## Highlights

- *What is already known on this topic:* The prevalence of STIs among FSWs varies across different countries and is determined by various sociodemographic factors, which are not known in Ethiopia.
- *What this study adds:* STIs are highly prevalent among FSWs in Ethiopia, and factors such as age, educational status, monthly income, and condom use determine their occurrence.
- *How this study might affect research, practice, or policy:* Awareness generation on the high prevalence and the determinants need to be considered in health policy and strategy formulation and further research on the prevention and control of STIs among FSWs.

## Introduction

Sexually transmitted infections (STIs) are common and constitute public health concerns globally (1, 2). They include viral infections such as human immunodeficiency virus (HIV), hepatitis B virus (HBV), and hepatitis C virus (HCV), as well as other bacterial STIs, specifically syphilis. According to the World Health Organization's (WHO) estimate, more than 1 million people are newly infected worldwide with STIs each day (2). This is equivalent to 374 million people infected per year, of which 96 million are in Africa. Globally, more than 4.5 million people contract HIV and viral hepatitis each year, and among adults aged 15 to 49, 7.1 million new cases of syphilis are identified (2–4).

STIs remain a major public health problem in Africa (5, 6). The Global Burden of Disease (GBD) (1) estimates that the age-standardized incidence rate (ASIR) for STIs is 9,535 per 100,000 person-years, with the highest rate estimated at 19,973 per 100,000 person-years in 2019 being in sub-Saharan Africa (SSA). STIs constitute the second-leading cause of mortality and disability-adjusted life years (DALYs) in low- and middle-income countries, particularly among those aged 20–24 years (3). Globally, over 2.3 million people died as a result of STIs (4) in 2021, which also accounted for the increased number of years of life lost (1). According to a study conducted in Botswana, DALYs increased with time due to HIV and other STIs (7).

STIs also increase the risk of cancer and account for 13% of global cancer incidence, with the highest rate in SSA in 2018 (4). HBV and HCV are the most common primary causes of cancer (8). STIs increase medical costs (9), are drug resistant, and are associated with an array of maternal and neonatal morbidities (3, 10), as well as a stigma among FSWs (11).

STIs are prevalent, particularly among key populations, including adolescents, young adults, and FSWs (4), owing to the high probability of co-infections, overlapping routes of transmission, and common health determinants. FSWs bear the greatest burden of STIs (4) because of their sexual behavior, which puts them at higher risk of acquiring the infection (1, 12); their vulnerability to violence and having the most limited access to health and social services exacerbate the problem (4).

The prevalence of STIs among FSWs varies by country and across different studies (11, 13–16) ranging from 13.3% (14) to 43.2% (11). In Ethiopia, 23% of FSWs reported having one or more STIs (15). The prevalence of STI co-infection in FSWs also varies across studies from different countries (14, 17–19). The prevalence of HIV and syphilis co-infection ranges across studies from 1.09 (14) to 43% (17), and HCV and HBV were reported in 40 and 2% of FSWs with HIV/AIDS worldwide, respectively.

The prevalence of STIs and co-infections is high among FSWs (9, 18, 20). Several factors determining the occurrence of STIs among FSWs have been reported, including age, lower education level (21), and unemployment (21, 22). Factors such as frequent unsafe intercourse with various sex workers (21, 23), current drug use, inconsistent condom use (24), HIV stigma (22), previous exposure to violence (25), lack of access to treatment, and the ability to pay for services (25) are among the most frequently identified determinants.

Concurrent multiple STIs occurring in individuals are often associated with having multiple sexual partners and can be a source of an STI epidemic among FSWs (26). In this population group, there is variation in STI co-infection prevalence and the associated determinants in different settings (14, 18, 19). Indeed, there is a need for further studies on the drivers of the number of STIs. The suggested STI categories (zero STI and at least one STI) in the data count used in previous studies might exhibit excess zero counts (no STI), and excluding the zero counts increases the possibility of biased estimates (27). In addition, counting data as non-negative might be over-dispersed and contain excess zeros, making data analysis complex (28). Therefore, in this analysis, we aimed to determine the factors that are associated with the number of STIs among FSWs in Ethiopia using robust statistical methods.

## Methods

### Study design, setting, and population

This study was a cross-sectional bio-behavioral study among FSWs conducted in 16 towns in Ethiopia between December 2019 and May 2020. The target towns include Adama, Addis Ababa, Arba Minch, Bahir Dar, Kombolcha/Dessie, Dilla, Dire Dawa, Gambella, Gonder, Harar, Hawassa, Jimma, Logia/Semera, Mizan, Nekemite, and Shashemane. We conducted respondent-driven sampling (RDS) on 6,085 FSWs aged 15 years and older who had received money or other benefits from selling sex to four or more people in the previous 30 days and had lived or worked in the surveyed town for at least the last month.

### Sampling and data collection

To recruit the participants, we employed respondent-driven sampling (RDS), a technique with benefits well-documented in previous reports (29, 30). As an initial step, 5 to 12 initial study participants referred to as “seeds” were selected from each study town. The seed participants were informed about the study, consent was obtained from each, and each participant was provided with three coupons for recruiting three eligible participants from her social network. All newly recruited participants were given three coupons as was done for the initial seeds to invite additional study participants. The data were collected through an anonymous interview administered by the study team in a private room using an Open Data Kit (ODK) electronic data management system with built-in skip patterns and logical validations.

### Study variables

The outcome variable in this study was the total number of STIs per FSW, categorized as zero STI, one STI, two STIs, three STIs, and four STIs. The independent sociodemographic variables included participants' age, the age at first sex sale, educational and married status, average monthly income, and the duration of sex work. Behavioral factors included alcohol and drug use, condom breakage, desired or forced first sex, HIV knowledge, and depression status.

The depression level was computed using the Patient Health Questionnaire (PHQ9) assessment tool (31). Participants with scores 0–4 were labeled as “non-minima”, 5–9 as “mild”, 10–14 as “moderate”, 15–19 as “moderately severe,” and 20–27 as “severe” depression severity levels.

Alcohol dependence level was computed using the alcohol use disorder identification test (AUDIT) (31). Drinking levels with scores 0–8 were labeled “social drinking”, 9–13 “harmful or hazardous drinking” for females, and 13–40 “alcohol dependence” severity levels.

Compressive HIV knowledge was computed from the four prevention and treatment and three misunderstanding knowledge questions. Respondents with seven true answers were labeled “had comprehensive knowledge” and otherwise “did not have comprehensive knowledge”.

### The testing procedure and quality control

The study used whole blood to test for HIV, HBV, HCV, and syphilis using a rapid diagnostic kit. HIV testing was done using the national algorithm, which included three rapid tests: assay 1 (STAT-PAK (HIV1/2, USA), assay 2 (ABONE, HIV1/2/O Tri-Line Device, Hangzhou, China), and assay 3 (SD Bioline, HIV1/2, USA). According to the algorithm, those who tested positive for all three were considered HIV-positive.

Hepatitis B surface antigen (HbsAg) detection was performed by the Virucheck HbsAg test kit manufactured in India. A one-step test for HbsAg detects the presence of HbsAg in serum or plasma specimens. Hepatitis C was screened using the Flaviscreen PLUSTM Test Kit produced in India. Flaviscreen is a rapid, third-generation, two-site sandwich immunoassay for the detection of total antibodies specific to the hepatitis C virus. It utilizes the principle of agglutination of antibodies or antisera with the respective antigen in the immunochromatography format.

Syphilis was screened using the Syphicheck-WB Screen and Confirm Assay produced in Kerala, India. Syphicheck is a rapid, qualitative immunoassay for the detection of antibodies to *Treponema pallidum*. It utilizes the principle of agglutination of antibodies in immunochromatography format. The interpretation of HBV, HCV, and Syphilis test results was according to the manufacturer's guide using the test kit insert.

The study staff received training to ensure the safety and effectiveness of the testing techniques, and standard operating procedures were followed throughout the process, including specimen collection, transportation, testing, and storage. The temperature was monitored while transporting the specimens, and invalid test results were repeated.

### Method of data analysis

Data were collected on tablet computers using the ODK software, exported to MS Excel, cleaned, and imported into STATA Version 16 for analysis. The RDS recruitment process (Tree of recruitment), RDS assumption assessment, and RDS weight generation were all carried out using the RDS package inbuilt into R statistical software (30, 32). Homophily and convergence, two common assumptions in RDS, were checked in HIV status, consistent condom usage, and FSW type and met the RDS criteria. The RDS weights were exported to STATA using the RDS-II function and merged with the entire dataset for further analysis. Descriptive statistics such as the crude and RDS-adjusted frequency, mean, and standard deviation were computed using RDS-II as a weighting variable. Univariable analysis was conducted to select potential risk factors to be considered in the final multivariable analysis using a modest level of significance (α = 0.25).

The Poisson, negative binomial, zero-inflated Poisson, zero-inflated negative binomial, hurdle Poisson, and hurdle negative binomial models were employed. The Poison regression model is a baseline count model for count data in which the variance of the dependent variable is equivalent to its mean (33). The deviance and Pearson's chi-square statistic values corresponding to their degree of freedom were used to test the presence of over-dispersion after fitting the Poisson regression model. In this case, the mean and variance were 0.27 and 0.53, respectively; thus, the assumption is violated, indicating that the data were dispersed. Then, a negative binomial regression model, the extension of the Poisson regression model was fitted to handle the problem of over-dispersion in the dataset (33). However, count data often exhibit an excess number of zeros (one cause of over-dispersion) which cannot be accommodated by the Poisson and negative binomial regression models (33). In the presence of zero inflation and over-dispersion, zero-inflated (zero-inflated Poisson and zero-inflated negative binomial) and hurdle models (hurdle Poisson and hurdle negative binomial) were frequently used to fit epidemiological data (27, 28, 33), and they provide a flexible and effective framework for modeling (33). The models have two parts: the first predicts non-zero STI counts (i.e., at least one STI), and the second predicts the zero-hurdle model (zero infections vs. not zero infections) among FSWs.

### Hurdle Poisson regression model

The hurdle Poisson regression model has two components: a truncated Poisson component with a rate parameter *u, u* > 0 that models non-zero positive counts, and a logit component with success probability, π_0_, π_0_ ≥ 0 that models the probability of zero counts. If the discrete random variable *Y*_*i*_ follows hurdle Poisson distribution, then the hurdle Poisson probability mass function is given as follows:


(1) $$\begin{array}{l} P\;(Y_{i}\;=\;y_{i}|\pi_{0},\text{μ})=\{\begin{array}{c} \pi_{0}\;,\;if\;y_{i}\;=\;0\\ \left(1-\pi_{0}\right)\frac{\exp(-\mu_{i})\mu_{i}{}^{y_{i}}}{y_{i}!(1-\exp(-\mu_{i}))},\;if\;y_{i}>0 \end{array} \end{array}$$

where 0 ≤ π_0_ ≤ 1, and defined by π_0_ = p (y = 0)

For the logit part, the conditional mean is given by E {*p*(*Y*_*i*_ = 0/*x*_*i*_)} = π_0_ (*x*_*i*_) = $\frac{\exp\left(X_{i}^{{}^{\prime }}\text{γ}\right)}{1+\exp\left(X_{i}^{{}^{\prime }}\text{γ}\right)}$. Taking natural logarithms in both sides of the equation, we have as follows:


(2) $$\begin{array}{l} \;logit\left(\pi_{0}\right)=\log\left(\frac{\pi_{0}}{1-\pi_{0}}\right)=Z^{{}^{\prime }}\\ \gamma =\gamma_{0}+\gamma_{1}X_{1}+\gamma_{2}X_{2}+\ldots \ldots ..+\gamma_{k}X_{k} \end{array}$$

where $\text{X}\;=\;{\left(1,\;X_{1},\;X_{2},\;\ldots \ldots ..,\;X_{k}\right)}^{{}^{\prime }}$ is a vector of independent variables, **γ** **=** $X\;=\;(1,\;X_{1},\;X_{2},\;\ldots \ldots ..,\;X_{k})\prime$ is a vector of regression coefficients, and log($\frac{\pi_{0}}{1-\pi_{0}}$) is the log transformation of the odds of at least one STI.

Similarly, the conditional mean for the truncated Poisson is given by: *E*(*Y*_*i*_/*x*_*i*_) = *u*_*i*_ = exp($X_{i}^{{}^{\prime }}\text{β}$)

Therefore, the truncated Poisson regression model is given by:


(3) $$\begin{array}{l} \log^{\left(u_{i}\right)}=X\prime \beta =\beta_{0}+\beta_{1}X_{1}+\beta_{2}X_{2}+\ldots ...+\beta_{k}X_{k} \end{array}$$

where $\text{X}\;=\;{\left(1,\;X_{1},\;X_{2},\;\ldots \ldots ..,\;X_{k}\right)}^{{}^{\prime }}$ and **β** **=** $\beta =(\beta_{0},\;\beta_{1},\;..,\;\beta_{k})\prime$ are a vector of independent variables and regression coefficients, respectively.

Each model's goodness-of-fit was evaluated using the Akaike Information Criteria (AIC), and rootogram visual assessment (34, 35). Both the count and the zero-inflated parts were analyzed. Finally, a 95% confidence interval (CI) was reported for the incident rate ratio and adjusted odds ratio. A *p*-value of ≤ 0.05 was used to define statistical significance.

### Ethical considerations

Ethiopian Public Health Institutes' Scientific and Ethical Research Office provided ethical clearance for the survey protocol (Ethical approval number: EPHI-IRB-108-2018). Each survey participant gave her consent to be interviewed, have blood specimens taken, and have the biospecimens stored for testing. Individuals who tested positive for STIs were transferred to the nearest or preferred health facility for appropriate clinical care. All collected information including the test results and seed contact information were kept entirely confidential.

### Patient and public involvement

Locally available organizations working on HIV prevention interventions, such as the HIV/AIDS Prevention and Control Office (HAPCO), District Health offices, and Drop-in Centers (DICs) were used to identify the initial participants (seeds) of the survey.

The seeds were selected based on the type of sexual worker, age category, and geographic location of the site. An FSW with a known social network was given three coupons so that she could invite her friends or other FSW contacts that were in her network. This approach allowed the study to reach as many eligible FSWs as possible. Finally, the findings of the study were shared with the FSW associations, HAPCO, and District Health offices through officially written letters and documents and using different platforms such as technical working group meetings and workshops.

## Results

### The magnitude of STIs

**Figure 1:**
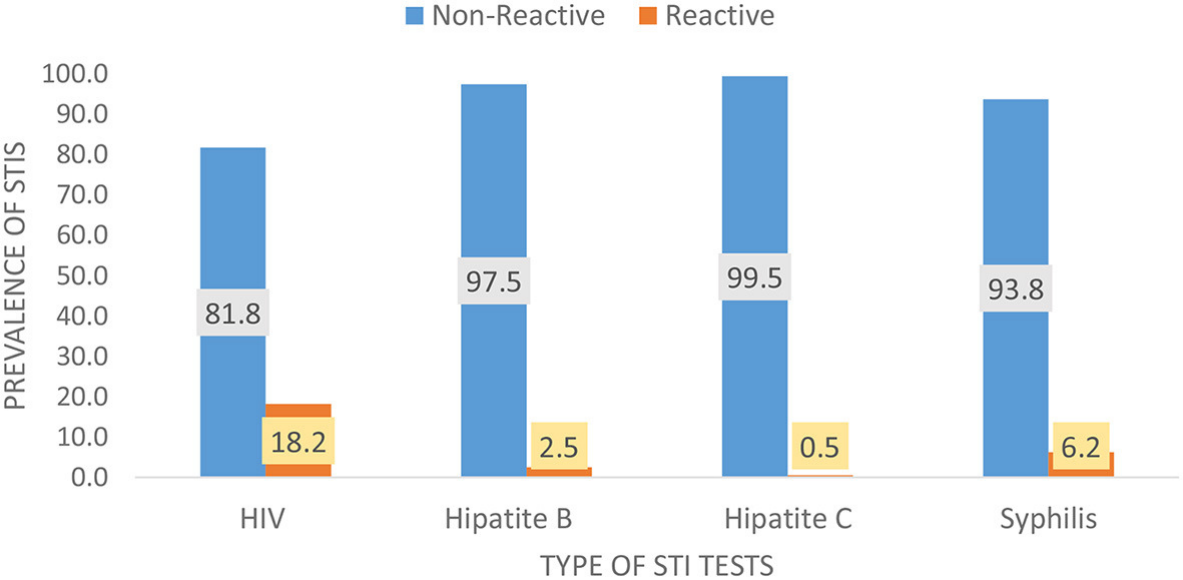
Weighted (RDS-adjusted) prevalence for the types of STIs among female sex workers in Ethiopia, bio-behavioral survey 2020.

**Figure 2:**
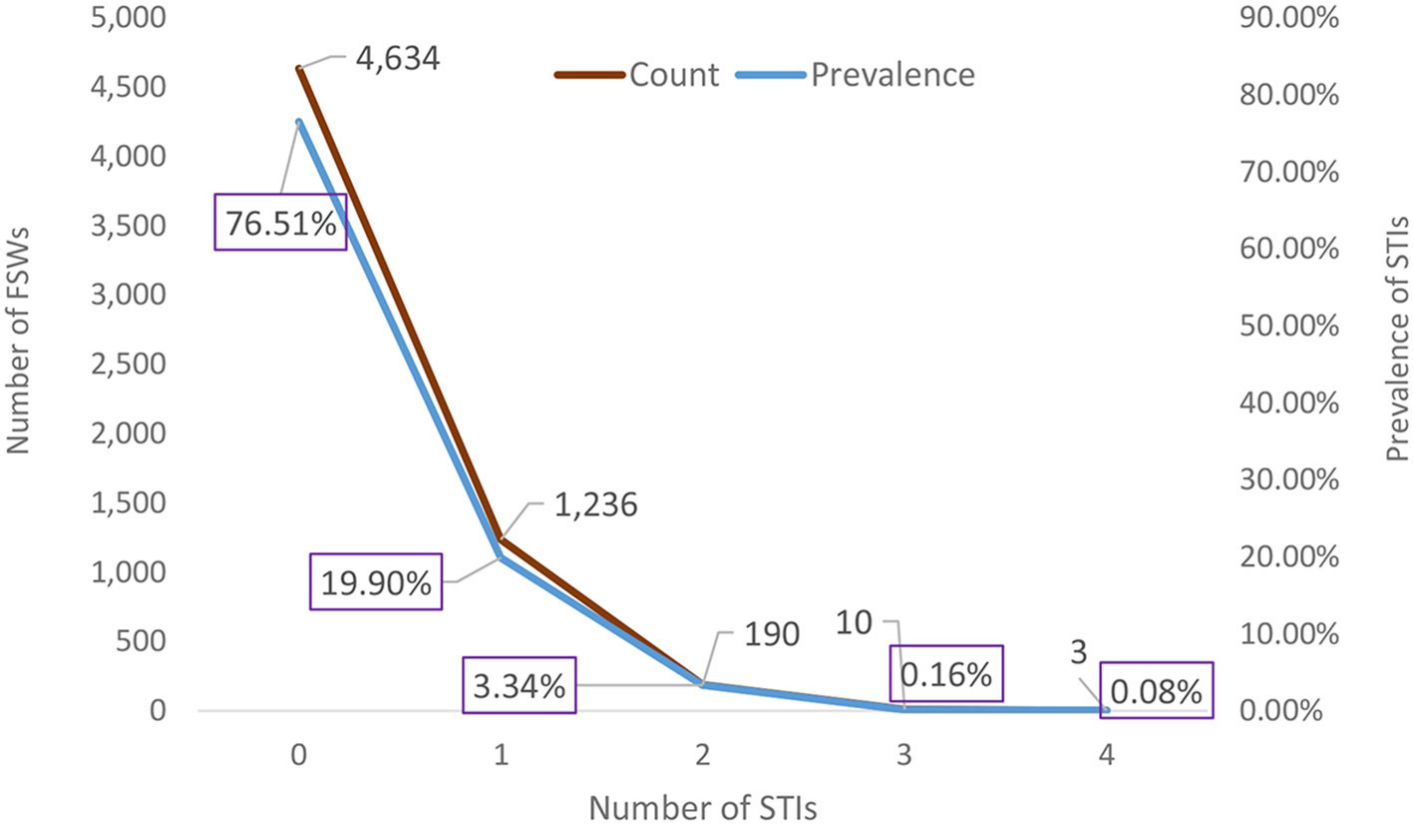
Weighted prevalence of the number of STIs among female sex workers in Ethiopia, bio-behavioral survey 2020.

Among the 6,085 FSWs involved in the study, 18.2% had HIV, 6.2% had syphilis, 2.5% had HBV, and 0.5% had HCV (Figure 1). Approximately one quarter, 1,439 (23.64%) of them, had at least 1 STI; 1,236 (19.90%) had 1 STI, 190 (3.34%) had 2 STIs, 10 (0.16%) had 3 STIs, and 3 (0.08%) had 4 STIs (Figure 2). The mean number of STIs among the FSWs was 0.27 [95% CI (0.26, 0.29)]. As shown in Tables 1, 2, comparing the mean number of STIs by category of the categorical covariates, on average, a higher number of STIs were observed among FSWs aged 35 years and above (0.64 ± 0.70), those who have a history of condom failure (0.34 ± 0.58), no formal education (0.45± 0.64), have moderate to severe depression (0.36±0.61), and are residing at Jimma (0.36 ± 0.62).

**Table 1:** Sociodemographic characteristics of female sex workers with sexually transmitted infections, bio-behavioral survey, Ethiopia, 2020 (N = 6,085).

**Characteristics**	**Crude value**		**Weighted value**	
	**n**	**%**	**Mean**	**Standard deviation**
**Study town**				
Adama	676	11.1	0.23	0.48
Addis Ababa	1,101	18.1	0.22	0.47
Arba Minch	251	4.1	0.33	0.57
Bahir Dar	372	6.1	0.35	0.56
Kombolcha/Dessie	251	4.1	0.32	0.58
Dilla	251	4.1	0.31	0.57
Dire Dawa	434	7.1	0.32	0.56
Gambella	468	7.7	0.26	0.47
Gonder	250	4.1	0.28	0.51
Harar	242	4	0.29	0.48
Hawassa	522	8.6	0.27	0.57
Jimma	254	4.2	0.36	0.62
Logia/Semera	251	4.1	0.26	0.51
Mizan	255	4.2	0.27	0.53
Nekemite	257	4.2	0.25	0.53
Shashemane	250	4.1	0.26	0.53
**Marital status**				
Married/cohabitation	231	3.8	0.28	0.54
Divorced/separated/ widowed	2,908	47.8	0.38	0.60
Never married	2,946	48.4	0.16	0.42
**The main source of income**				
Sex work	5,694	93.6	0.27	0.52
Other than sex work	391	6.4	0.38	0.62
**Average monthly income from selling sex in USD**				
<65	1,778	29.2	0.37	0.60
65 and 150 USD	2,066	34	0.27	0.52
150 to 200 USD	1,175	19.3	0.23	0.48
200 and above USD	1,066	17.5	0.17	0.43
**Age at first sex selling**				
<20	2,328	38.3	0.18	0.44
20–24	2,348	38.6	0.25	0.51
25+	1,406	23.1	0.46	0.62
**Age at first sex**				
15 or less	2,430	39.9	0.33	0.56
16–20	3,384	55.6	0.23	0.49
21+	271	4.5	0.31	0.54
**Age category**				
15–24	2,595	42.6	0.11	0.35
25–34	2,671	43.9	0.32	0.54
35–59	819	13.5	0.64	0.70
**Highest educational status attained**				
Non-formal education	1,054	17.3	0.45	0.64
Primary school (grades 1–8)	3,560	58.5	0.26	0.51
Secondary school and above	1,471	24.2	0.18	0.44
**Year lived as SFWs**				
<5 Years	2,307	38	0.18	0.43
5–10 Years	1,556	25.6	0.31	0.54
11+ Years	2,110	36.4	0.35	0.58

**Table 2:** Behavioral and clinical characteristics of female sex workers with sexually transmitted infections, bio-behavioral survey, Ethiopia, 2020 (N = 6,085).

**Characteristics**	**Crude value**		**Weighted values**	
			**Mean**	**Standard Deviation**
**Level of depression**				
Not depressed	2,468	40.6	0.24	0.50
Mild depression	2,525	41.5	0.27	0.51
Moderate to severe depression	1,092	17.9	0.36	0.61
**Alcohol drinking (AUDIT)**				
Social drinking/not risky	2,594	42.8	0.29	0.56
Harmful or hazardous drinking	1,210	20	0.27	0.51
Alcohol dependence indication	2,257	37.2	0.26	0.50
**Chewing khat in the last 30 days**				
Never	2,258	37.1	0.28	0.54
Yes	3,827	62.9	0.27	0.52
**Alcohol use**				
Never	5,385	88.5	0.28	0.53
Yes	700	11.5	0.25	0.53
**First sex experience**				
Wanted	4,738	77.9	0.25	0.51
Forced	1,347	22.1	0.34	0.59
**Changed location of selling sex in the past 6 months**				
No	4,572	75.1	0.27	0.52
Yes	1,513	24.9	0.29	0.54
**Number of cities worked sex selling in the last 3 years**				
Same town	4,933	81.1	0.27	0.52
1 more town	776	12.8	0.27	0.52
2 or more towns	374	6.1	0.28	0.57
**Having sex without a condom in the last 30 days (at least once)**				
Yes	5,119	84.1	0.31	0.58
No	966	15.9	0.27	0.51
**Experienced condom failure in the last 30 days**				
No	4,260	70	0.25	0.50
Yes	1,825	30	0.34	0.58
**Ever had anal sex**				
Never	5,659	93	0.28	0.53
Yes	426	7	0.23	0.49
**Number of non-paying partners in the past 6 months**				
Never	4,347	71.4	0.28	0.53
Only one	1,404	23.1	0.26	0.53
2 and more	334	5.5	0.30	0.50
**At least two STI symptoms occurred in the last 12 months**				
No	5,083	83.5	0.26	0.52
Yes	1,002	16.5	0.33	0.56
**Ever been raped or forced to have sex in the past 12 months**				
No	5,314	87.3	0.27	0.53
Yes	771	12.7	0.30	0.53
**Number of pregnancies**				
0	1,873	30.8	0.16	0.41
1	2,059	33.8	0.25	0.51
2	1,238	20.3	0.34	0.57
3+	915	15	0.47	0.64
**Currently pregnant**				
No	5,973	98.2	0.28	0.53
Yes	112	1.8	0.15	0.43
**History of miscarriage pregnancy**				
No	5,471	89.9	0.26	0.52
Yes	614	10.1	0.38	0.59
**History of aborted pregnancy**				
No	4,809	79	0.27	0.52
Yes	1,276	21	0.29	0.53
**Number of clients in the past 6 months**				
4–30	2,308	37.9	0.31	0.56
31–90	2,132	35	0.26	0.51
91+	1,645	27	0.24	0.49

### Model selection

The distribution of the number of STIs is skewed to the right, signifying the likelihood of over-dispersion. The zero STIs on the bar charts in Figure 2 are highly selected, suggesting that count data models that account for excess zeros, such as zero-inflated models and hurdle models, would better fit the data of the number of STIs. The hurdle Poisson model has the smallest AIC value and is considered the final model (see Table 3). Furthermore, a visual assessment of the fit was made in terms of the rootogram.

**Table 3:** Model selection and comparison of the number of STIs among female sex workers.

**Model selection**	**Poisson**	**Negative binomial**	**Zero-inflated Poisson**	**Zero-inflated negative binomial**	**Hurdle Poisson**	**Hurdle negative binomial**
AIC	7,139.42	7,141.49	7,146.22	7,148.22	7,093.25	7,095.25
Log-likelihood	−3,522	−3,531	−3,537	−3,537	−3,511	−3,511

**Figure 3:**
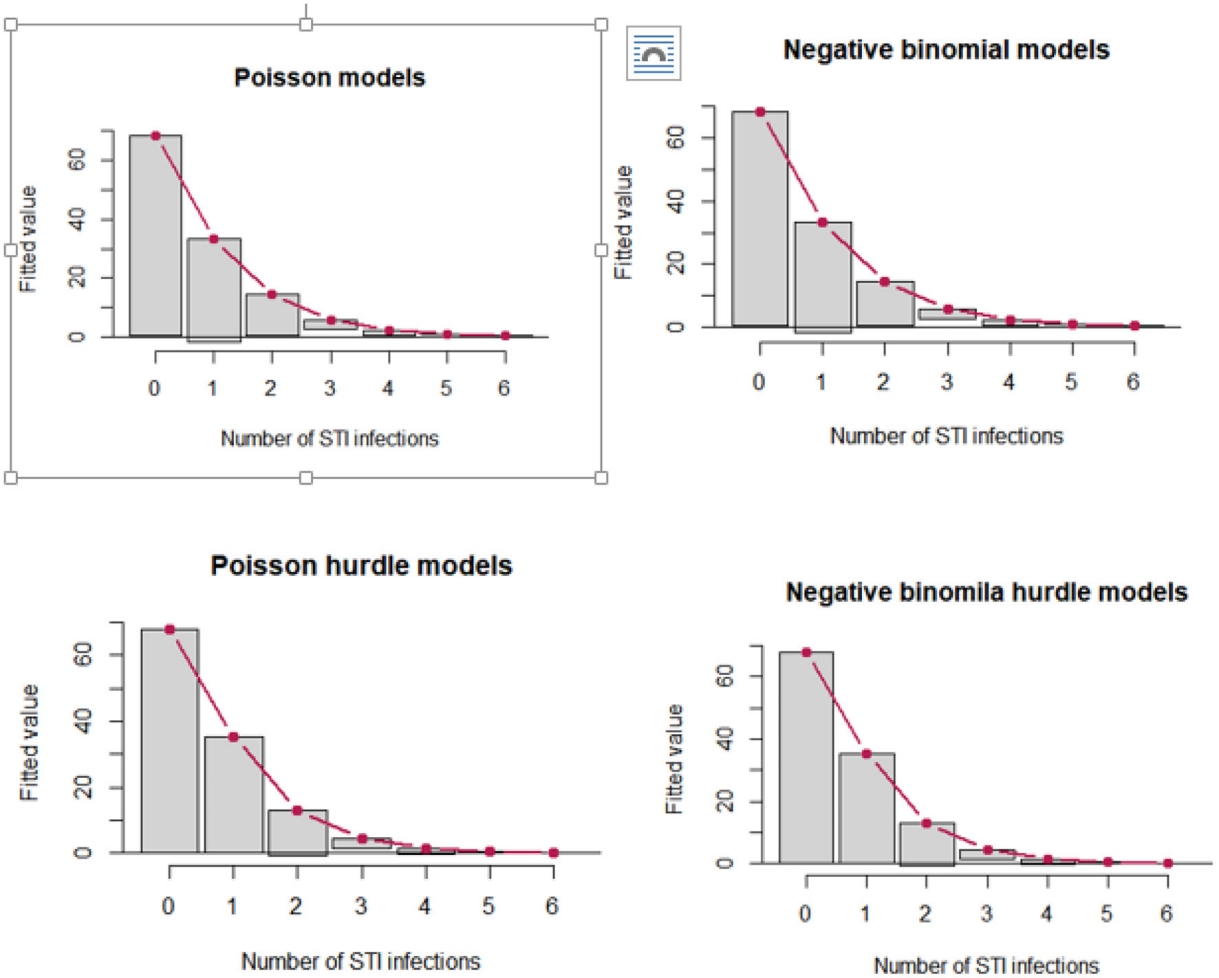
Hanging rootograms for count regression models of STIs among female sex workers in Ethiopia, bio-behavioral survey 2020.

Hurdle negative binomial has an inferior fit, with some low numbers over-predicted. The hurdle Poisson model was found to be the best fit for the data based on their respective log-likelihood, AIC, and rootogram (Figure 3).

### Factors associated with the number of STIs

The model is divided into two sections (Table 4): the first predicts non-zero counts of STIs (truncated negative binomial with log link), and the second predicts the zero-hurdle model (binomial with logit link) with zero STIs vs. no zero STIs.

**Table 4:** Factors associated with the number of sexually transmitted infections (STIs) among female sex workers, bio-behavioral survey, Ethiopia, 2020.

**Characteristics**	**Count model coefficients (Truncated Poisson with log link)**			**Zero-hurdle model coefficients (binomial with logit link)**		
		**Incidence rate ratio**	**Std. err**	**95% CI**	**AOR**	**Std. err**	**95% CI**
**Age**	15–25 (ref)						
	25–34	1.45	0.34	0.91, 2.3	2.99	0.25	2.54, 3.52^**^
	35–59	2.32	0.57	1.43, 3.74^*^	8.05	0.85	6.54, 9.91^**^
**Educational status**	Non-formal education (ref)						
	Primary school (1-8)	0.88	0.13	0.66, 1.17	0.69	0.06	0.59, 0.81^**^
	Secondary school and above	0.8	0.18	0.52, 1.23	0.52	0.05	0.42, 0.64^**^
**Average monthly income**	<65 (ref)						
	65 and 150 USD	0.86	0.13	0.64, 1.16	0.70	0.06	0.60, 0.82^**^
	150 to 200 USD	0.83	017	0.55, 1.23	0.68	0.07	0.56, 0.82^**^
	200 and above USD	0.74	0.19	0.44, 1.24	0.51	0.06	0.42, 0.63^**^
**Depression**	No depression (ref)						
	Mild depression	0.97	0.16	0.70, 1.34	1.04	0.08	0.90, 1.20
	Moderate to severe	1.55	0.26	1.12, 2.18^*^	1.21	0.11	1.01, 1.45^**^
**Drug use–AUDIT score**	Social drinking/not risky (ref)						
	Harmful/hazardous drinking	0.81	0.14	0.58, 1.15	1.06	0.09	0.89, 1.27
	Alcohol dependency indication	0.62	0.10	0.45, 0.86^*^	1.06	0.08	0.91, 1.24
**First sex forced**	Wanted (ref)						
	Forced	1.32	0.19	1.01, 1.74^*^	1.13	0.09	0.97, 1.32
**Age at first sex**	≤ 15 (ref)						
	16–20	0.98	0.13	0.75, 1.28	0.72	0.05	0.63, 0.83^**^
	≥20	0.74	0.25	0.37, 1.45	0.67	0.10	0.50, 0.91^**^
**Year lived as FSWs**	≤ 5 (ref)						
	5–10	1.03	0.21	0.70, 1.52	1.30	0.11	1.10, 1.55^**^
	≥11	1.10	0.20	0.76, 1.58	1.21	0.10	1.03, 1.43^**^
**Condom break**	No (ref)						
	Yes	1.32	0.18	1.01, 1.74^*^	1.40	0.09	1.21, 1.61^**^

^*^Statistically significant Incidence rate ratio; ^**^Statistically significant Adjusted odds ratio (AOR).

#### Truncated Poisson with log link function

Table 4 shows the results of hurdle Poisson model parameter estimates, incidence rate ratio (IRR), standard error, *P*-values, and 95% CIs for IRR. In the results of a truncated Poisson with a log link function to predict the number of at least one STI found, after controlling for the effect of other characteristics, FSWs' age, condom breakage, a history of moderate to severe depression, and drug use were significant predictors of STIs.

When compared to FSWs under the age of 25 years, those aged 35–49 were approximately 2.3 times [IRR = 2.3; 95% CI (1.43, 3.74)] more likely to experience STIs. FSWs with moderate to severe depression were 1.55 times (IRR = 1.554; 95 % CI: 1.15, 2.18) more likely to have STIs compared to those without depression.

FSWs who were forced to have sex at their first sexual encounter were 1.32 times [IRR = 1.32; 95% CI (1.01, 1.74)] more likely to have STIs compared with those who willingly had sex at their first encounter. When compared to FSWs who had not experienced condom breakage during sexual intercourse, those who had experienced condom breakage were 1.32 times [IRR = 1.32; 95% CI (1.01, 1.74)] more likely to have STIs.

The estimated frequency of STIs experienced by an FSW was significantly associated with the alcohol dependency indicator. Compared to social drinking/not risky FSWs, those with alcohol dependency signs had a 37.7% (IRR = 0.62; 95% CI: 0.45, 0.86) lower risk of STIs.

#### Zero-hurdle model (binomial with logit link)

The second predicts the zero-hurdle model (binomial with logit link) with zero STIs vs. no STIs. The zero-hurdle model's estimated adjusted odds ratio (AOR) and 95% CI for the factor change in the odds of experiencing at least one STI are shown in Table 4. After controlling for all other factors in the model, the probability of having an STI was found to be significantly associated with age, education level, average monthly income, depression, age at selling sex, years spent as FSWs, and condom breakage.

From the results of the hurdle model (binomial with logit link**)**, the odds of having STI (at least one STI) were lower by 31% [AOR = 0.69; 95% CI (0.59, 0.81)] and 48% [AOR = 0.52; 95% CI (0.42, 0.64)] for FSWs who had attended primary and secondary school or above, respectively, compared to those who had no formal education. FSWs aged 25–34 and 35–59 years had approximately three times [AOR = 2.99; 95% CI (2.54, 3.52)] and eight times [AOR = 8.051; 95% CI: (6.54, 9.91)] the chances of having at least one STI, respectively, compared to FSWs aged 24 years or younger.

FSWs earning between 65 and 150 USD, 150 to 200 USD, and 200 and above USD had a 30% [AOR = 0.70; 95% CI (0.60, 0.82)], 33% [AOR = 0.68; 95% CI (0.56, 0.82)], and 49% [AOR = 0.51; 95% CI (0.42, 0.63)] lower risk of having at least one STI. Those who began selling first sex between the ages 16–20 years and at the age of 20 or more had a 28% [AOR = 0.72; 95% CI (0.63, 0.83)] and 33% [AOR = 0.67; 95% CI (0.50, 0.91)] lower risk of experiencing at least one STI than those who began selling first sex at the age of 15 years below. FSWs who worked on this business for <5 years were 1.3 [AOR = 1.3; 95% CI (1.1, 1.55)] and 1.21 [AOR = 1.21; 95% CI (1.03, 1.43)] times more likely to have at least one STI.

FSWs with a history of condom breakage were 1.4 [AOR = 1.4; 95% CI (1.21, 1.61)] times more likely to have at least one STI than those without a history of condom breakage. The odds of experiencing at least one STI were 1.21 [AOR = 1.21; 95% CI (1.01, 1.45)] times higher in FSWs who were moderately or seriously depressed compared to those who did not have depression.

## Discussion

This study found that 18.2% of the 6,085 FSWs studied had HIV, 6.2% had syphilis, 2.5% had HBV, and 0.5% had HCV. This study found that at least one STI was found in 23.64% of the FSWs, which is consistent with the findings of previous reports from Ethiopia (15), Mexico (16), and Ecuador (13) where at least 17.6% of FSWs were infected with STIs. Our finding could be a higher estimate because the other studies used polymerase chain reaction (PCR) to test for STIs.

The finding among the FSWs in our study is higher than those of the reports from Brazil of 13.3% (14), but lower than the Russian report (11), which showed that 43.2% of participants had at least one STI. This disparity could be due to differences in sociodemographic characteristics, STI diagnostic methods, types, and the number of STIs included in the specific studies.

Age, educational status, and average monthly income, as well as a history of depression, condom breakage, early initiation of sex selling, and living as a sex worker for a long time were associated with the frequency of STIs in our study. This suggests that the country has to make a greater effort to work toward increasing awareness among FSWs as well as the general population and improving prevention, care, and treatment services for STIs among FSWs.

Our study found that FSWs over the age of 30 were more likely to have at least one STI. This finding is comparable to those reported by studies conducted in Namibia (21), the Republic of Congo (29), South Africa (36), Ecuador (13), and Rwanda (19). Studies show an increase in STIs and co-infections with age, probably due to older FSWs not considering themselves high risk and failing to persuade customers to use condoms (13, 20). This could also be due to the cohort effect, where older women have more chances of acquiring STIs. Consequently, FSWs in this age group could be the major drivers of STIs among FSWs and their clients unless effective and comprehensive programs are implemented. In contrast, a report from Iran indicates that being under the age of 25 is independently associated with increased STIs (37). This disparity could be associated with sociocultural differences between population groups, but it requires further exploration.

Similar to a finding in Rwanda (19), FSWs who were in the business for a long time in our study had higher odds of getting at least one STI. Similarly, those who began selling sex at a young age had the highest odds of having at least one STI, observations also reported from Iran (37). This may be explained by the fact that young sex workers are more likely to report inconsistent condom use and condomless sex with their last clients (38, 39). FSWs who started the business at earlier ages are also more likely to drink alcohol heavily (40) and experience multiple clients each day (41). However, these findings contrast the Rwandan study (38), which found no statistically significant association between sex work starting at earlier ages and STIs.

Our finding shows that, compared to those who had no formal education, the odds of experiencing at least one STI was lower among educated FSWs is consistent with findings from the studies in Rwanda (19), Russia (11), and Namibia (21). As reported by others (21), this could be because those with low educational levels are more likely to be unemployed and engage in risky sexual behaviors associated with STIs (42). Similarly, FSWs with low average monthly income were more likely to experience at least one STI in our series, which could be because the sex workers earned less money as they got older and had to do sex work more often with many clients, and the clients decided to use condoms (41).

Condom breakage increases the frequency of STIs and increases the probability of having STIs among FSWs as shown by our study, which identified that those with a history of condom breakage had an increase in the rate of developing at least one STI by 46%. This may be because those experiencing condom breakage were exposed to pornography and used sexual enhancement products (43). The finding is consistent with previous reports for Ethiopia (15) and China (44). Available evidence has also shown that consistent and high levels of condom use among FSWs decrease the incidence of STIs among sex workers as well as the general population.

## Strengths and limitations

Overall, the advantage of our study, which was a nationwide survey involving a large sample of FSWs recruited by using the RDS technique from 16 towns across the country, outweighs a venue-based selection approach in terms of obtaining a representative sample. However, as this was a cross-sectional study, temporal relationships between determinants and the outcome cannot be established. In addition, key measures rely on self-report; biases such as social desirability response bias could have some effect. The rapid test/serological markers we used in this survey do not detect the duration of infections as recent or long-term. In this study, we only considered HIV, HBV, HCV, and syphilis, our results could underestimate the overall prevalence of STIs.

## Conclusion

The prevalence of STIs among FSWs is high in Ethiopia. Age, educational status, average monthly income, history of depression, history of condom breakage, early initiations of selling sex, and living as FSWs for a long period of time are identified as independent predictors for developing at least one STI. Targeted STI prevention and control programs need to be improved, with a focus on promoting higher education among women, condom distribution, and the creation of awareness on proper and consistent use, enhancing STI testing, prevention, care and treatment, interventions, and supporting income-generating activities. Further epidemiological research is needed on STIs among FSWs in Ethiopia to determine the magnitude of the problem, which should include a broader list of STIs, confirmatory diagnostic tests, and recency testing.

## Data availability statement

The raw data supporting the conclusions of this article will be made available by the authors, without undue reservation.

## Ethics statement

The studies involving human participants were reviewed and approved by PHI-IRB-108-2018 and the Ethiopian Public Health Institute. The participants provided their written informed consent to participate in this study.

## Author contributions

FW, SA, JA, SL, and BB conceived the manuscript. FW wrote the first draft and with further contributions from all authors. JA and DA conducted the statistical analysis with support from FW, BB, JT, AB, MR, and SA. JA and DA undertook data management. LN, GE, WB, MA, FW, and SA are principal investigators (PIs). FW was the guarantor of the manuscript, accepted full responsibility for the study, had access to the data, and controlled the decision to publish. All authors contributed to data interpretation, reviewed successive drafts, and approved the final version of the manuscript.
